# Novel insights from non-conserved microRNAs in plants

**DOI:** 10.3389/fpls.2014.00586

**Published:** 2014-10-28

**Authors:** Zhengrui Qin, Chunlian Li, Long Mao, Liang Wu

**Affiliations:** ^1^National Key Facility for Crop Gene Resources and Genetic Improvement, Institute of Crop Science, Chinese Academy of Agricultural Sciences, Beijing, China; ^2^State Key Laboratory of Protein and Plant Gene Research, College of Life Sciences, Peking University, Beijing, China

**Keywords:** miRNA, role, secondary siRNA, dicots, monocots

## Abstract

Plant microRNAs (miRNAs), a class of small non-coding regulatory RNAs, are canonically 20–24 nucleotides in length and bind to complementary target RNA sequences, guiding target attenuation via mRNA degradation or translation inhibition. Of the annotated miRNA families, evolutionarily conserved families have been well known to extensively regulate analogous targets and play critical roles in plant development and adaptation to adverse environments. By contrast, majority of these families that are merely present in a specific lineage or in a few closely related species have not been well functionally explored until recently. The fast-growing progresses being made in the actions of non-conserved miRNAs nowadays in diverse plant species may represent a highly promising research field in future. This review thereby summarizes the emerging advances in our understanding of the biogenesis, associated effectors, modes to targets, and biological functions of plant non-conserved miRNAs. In addition, it outlines the regulatory units recently discovered between conserved miRNAs and their alternative targets.

## INTRODUCTION

In the past decade, small silencing RNAs (sRNAs) have been identified as key components of gene modulatory networks in eukaryotes. In plants, based on their origins, structures, and actions on targets, sRNAs are classified into two major categories: small interfering RNAs (siRNAs) and microRNAs (miRNAs; Meyers et al., 2008). Even though the diverse sets of siRNAs, including heterochromatic siRNAs (hc-siRNAs), which are also named repeat-associated siRNAs (ra-siRNAs), trans-acting siRNAs (ta-siRNAs) and natural antisense transcript-derived siRNAs (nat-siRNAs) have similar origination from double-stranded transcripts, their actions on targets are largely dependent on their sizes, for instance, 21-nucleotide (21-nt) siRNAs usually cleave target mRNAs for post-transcriptional gene silencing (PTGS) while 24-nt siRNAs guide target chromatin remodeling through a mechanism called RNA-directed DNA methylation (RdDM) mediating transcriptional gene silencing (TGS; Allen and Howell, 2010; Law and Jacobsen, 2010).

Compared with siRNAs, the criteria for defining miRNAs is based on the distinctive nature of miRNA biogenesis. miRNAs come from pre-miRNAs that adopt canonical stem-loop transcripts (Meyers et al., 2008). One important characteristic of miRNA distinct from siRNA is that the sequence of mature miRNA has little heterogeneity from other small RNAs in its hairpin precursor structure. With the powerful capacity of high-throughput sequencing technologies and development of bioinformatics prediction approaches, there has been a dramatic explosion in the number of miRNAs reported in recent years. In most cases, plant miRNAs are 20- to 22-nt in length and mediate target regulation via mRNA digestion or protein translation inhibition. In particular, 24-nt long miRNAs (lmiRNAs) in rice are produced and are able to serve as epigenetic regulators to targets in a DNA methylation manner (Wu et al., 2010), suggesting complicated miRNA populations and function manners in plants.

## CONSERVATION OF miRNAs IN PLANTS

According to the conservation and diversification of miRNAs during evolution in the plant kingdom, miRNA families can be classified to two different categories: the ancient miRNAs and the young miRNAs (Axtell and Bowman, 2008; Tang, 2010; Cuperus et al., 2011). The ancient miRNAs are often highly expressed and evolutionally conserved, while the young miRNAs are expressed lowly or only induced by specific conditions and generally exist only in limited species, resulting in being evolutionarily non-conserved (Cuperus et al., 2011; Taylor et al., 2014).

### CONSERVED miRNAs

Green plants form a clade of eukaryotes that includes green algae, a single-cell organism, and living multicellular organisms, including *Cyanophora* and Embryophyta. Embryophyta is the most populous subkingdom of green plants formed vegetation on earth, containing mosses, ferns, gymnosperms, and angiosperms (Taylor et al., 2014). Although there are no strictly conserved miRNAs among all organisms with chloroplast because none of the same miRNAs have been identified from single-celled algae *Chlamydomonas* and multicellular plants (Molnar et al., 2007; Zhao et al., 2007), several ancient miRNAs such as miR156, miR160, miR165/166, miR167, miR319, miR390, miR395, and miR408 are present across land plants as well as in the non-flowering moss, suggesting that these miRNA families are universal in Embryophyta lineages (Taylor et al., 2014). In this review, we designated universal families with identification numbers from miR156 to miR408 that are common between gymnosperms and angiosperms including eudicotyledons (dicots) and monocotyledons (monocots) as conserved miRNAs (Cuperus et al., 2011; Taylor et al., 2014). To date, the known biological significance of plant miRNAs are primarily from those conserved miRNAs, which predominantly regulate ancestral transcription factors or physiological enzymes involved in basic plant development or tolerance to stresses.

### NON-CONSERVED miRNAs

A large number of plant miRNAs and their corresponding targets are only present within a few closely related species or appear to be unique to specific species. These lineage-specific miRNAs are customarily called non-conserved miRNAs so as to distinguish them from the conserved miRNAs. Unlike conserved miRNAs with high abundance and low sequence variations, non-conserved miRNAs are often processed imprecisely, expressed weakly, and lack of functional targets; thus in some cases they have been considered as transient products and energy wasters in the plant genome. Despite this, some non-conserved miRNAs are expressed abundantly in specific tissues or greatly induced under particular conditions, supporting a possible physiological role of plant non-conserved miRNAs for special environmental adaptations. Efforts to understand lineage-specific miRNAs in different plants will help elucidate how these young miRNAs affect special growth processes.

## BIOGENESIS, ASSOCIATED EFFECTORS, AND ACTIONS OF miRNAs IN PLANTS

Research on plant miRNA biogenesis and functions was beginning from the highly conserved miRNAs. Genetic conclusions obtained from *Arabidopsis* and rice firstly have shown that conserved miRNAs share the same machinery in transcription, processing and action fashions. Later on, along with the development of high-throughput sequencing approaches and the resultant produced datasets, people identified more and more lowly expressed and evolutionarily novel miRNAs. Hence, studies on the biogenesis and function pathways of non-conserved miRNAs have been given more and more attentions in recent years.

### BIOGENESIS OF CONSERVED miRNAs

During long-time evolution history, conserved miRNAs are restricted to similar biogenesis and action machinery. Like protein-coding genes, pri-miRNAs are transcribed by RNA polymerase II (Pol II) and are modified at post-transcriptional layers, occupying a 5′ cap and a 3′ poly (A) tail. But unlike that of in animals, which have diverse origins, plant primary miRNAs are usually transcribed from intergenic regions. The key processor for conserved miRNA precursor and duplex in plant is an RNA endoribonuclease, namely Dicer-like 1 (DCL1), working in a slicing complex comprising double-stranded RNA-binding protein HYPONASTIC LEAVES1 (HYL1), C2H2-zinc finger protein SERRATE (SE), and nuclear cap-binding complex (CBC; Dong et al., 2008; Laubinger et al., 2008). After methylation at the 3′-end by HUA ENHANCER 1 (HEN1; Yu et al., 2005), the mature miRNA duplexes are transported into the cytoplasm from the nucleus by plant exportin 5 ortholog HASTY (Park et al., 2005). Subsequently, one strand called passenger strand is degraded while the other strand called guide stand is recruited into ARGONAUTE1-containing RNA-induced silencing complexes (RISCs; Park et al., 2005). These RISCs silence target genes through mRNA degradation or protein translation repression based on RNA–RNA base-pairing (Baumberger and Baulcombe, 2005; Qi et al., 2005; Brodersen et al., 2008).

In addition to sRNA loading and RISCs activities, some AGO proteins such as AGO2 in animal cells have been implicated in increasing mature miRNA levels by feedback mediating miRNA processing and stabilization (Winter and Diederichs, 2011). We are not going to discuss the conserved miRNA pathway in detail here because excellent descriptions on biogenesis and action nature of conserved plant miRNAs have been available in several recent reviews (Voinnet, 2009; Chen, 2012; Rogers and Chen, 2012, 2013; Wu, 2013).

### BIOGENESIS OF NON-CONSERVED miRNAs

Although some non-conserved miRNAs are transcribed from gene exon or intron regions, the majority of them still originate from intergenic loci similar to conserved miRNAs. However, different from those of conserved miRNAs, which occupy multiple copy numbers from several DNA loci, primary transcripts of non-conserved miRNAs are usually transcribed from relatively less genomic regions and are processed by diverse DCLs, thus probably generating some heterogeneous small RNA products in the precursors. Although the major product is miRNA, other processed-sRNAs from the same precursor may also enter alternative RISCs and could be conferred different functions to act on targets (Rajagopalan et al., 2006; Vazquez et al., 2008; Voinnet, 2009).

**FIGURE 1:**
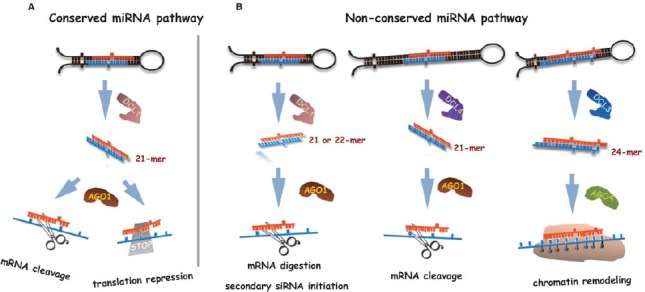
**A model for biogenesis and actions of conserved and non-conserved miRNAs in plants. (A)** Conserved miRNAs are usually generated by DCL1 and are specifically loaded into AGO1 to guide the cleavage of their target mRNAs or mediate translation inhibition of target proteins. **(B)** Lineage-specific miRNA with a short hairpin precursor (left) is typically processed by DCL1 and is associated with AGO1 complex. Among them, 21-nt miRNAs cleave the target mRNA while 22-nt miRNAs initiate secondary siRNA formation in addition to mRNA digestion for target regulation enhancement. Some non-coding RNA transcripts with long stem-loop structures (middle) are processed by DCL4 rather than DCL1. The resultant 21-nt non-conserved miRNAs subsequently recruited to AGO1 and guide target mRNA degradation. The 24-nt long miRNAs (right) are processed by DCL3 from their precursors and are sorted into AGO4 to methylate DNA at target gene loci via interaction with other components in RNA-directed DNA methylation (RdDM) pathway.

In contrast with conserved miRNAs, which are characterized to be universally processed by DCL1, the identified non-conserved miRNAs could be produced by distinct DCLs (Figure 1B). Both DCL4 and DCL1 can catalyze the cleavage of RNA transcripts into 21-nt fragments, but DCL4 is originally known to produce endogenous phased and exogenous virus-induced siRNAs (Henderson et al., 2006). Genetic analysis from *Arabidopsis* and rice has found that several non-conserved miRNAs, especially those with long hairpin precursor structures, were absent from *dcl4* but not *dcl1* mutants, such as miR822 and miR839 in *Arabidopsis* and miR7695 in rice, indicating that DCL4 rather than DCL1 is required for these 21-nt non-conserved miRNA biogenesis (Figure 1B; Rajagopalan et al., 2006; Campo et al., 2013).

DCL3 is the only Dicer-like protein that routinely generates 24-nt long sRNA fragments required for hc-siRNA formation in plants. Although most plant miRNAs are 21-nt (20 ∼ 22 nt) in length, a class of 24-nt (23–25 nt) lmiRNAs that depend on DCL3 has been recently identified in rice and *Arabidopsis* (Vazquez et al., 2008; Chellappan et al., 2010; Wu et al., 2010). In contrast to 21-nt miRNAs acting on targets through AGO1-silencing complex at the post-transcriptional level, lmiRNAs are predominantly associated with AGO4 clade proteins which have been originally discovered as essential effectors in RdDM pathway repress targets via DNA methylation (Wu et al., 2010). Interestingly, a subset of pri-miRNAs in rice have been found processed by the coordinated action of DCL1 and DCL3 to simultaneously produce both 21-nt miRNAs and 24-nt lmiRNAs that are hierarchically bound by AGO1 and AGO4 complexes, respectively (Wu et al., 2010). In this regard, a pri-miRNA may have dual functions, producing two miRNA variants that guide either mRNA digestion or chromatin remodeling. Therefore, it is reasonable to think that the 24-nt miRNAs are subjected to short-term selection and evolution, thus improperly entering into the hc-siRNA processing pathway (Figure 1B; Table 1; Wu et al., 2010).

**Table 1:** **Biogenesis and action pathways of conserved and non-conserved miRNAs in plants.**

miRNA type	Self-complementary status of precursor	Biogenesis	Size (nt)	RISCs	Modes to targets	Example	Reference
Conserved	Imperfect	DCL1	21	AGO1	mRNA cleavage, translation inhibition	172, 319	Palatnik et al. (2003), Chen (2004)
Non-conserved	Less imperfect	DCL1	21	AGO1	mRNA cleavage	824, 5200	Kutter et al. (2007), Wu et al. (2013)
	Less imperfect	DCL1	22	AGO1	mRNA cleavage, phased-siRNA initiation	173, 6019	Montgomery et al. (2008), Li et al. (2012)
	Less imperfect	DCL4	21	AGO1	mRNA cleavage	822, 7695	Rajagopalan et al. (2006), Campo et al. (2013)
	Less imperfect	DCL3	24	AGO4	DNA methylation	1863, 1884	Wu et al. (2010)

DCL2 is a special DCL protein essential for 22-nt sRNA production. In *Arabidopsis* *dcl3* mutants, the 24-nt long miR825, miR826, and miR827 are replaced by 22-nt miRNA species that are not present in wild-type or other *dcl* mutants, implying a potential role of DCL2 in processing certain pri-miRNAs to generate 22-nt miRNAs (Vazquez et al., 2008).

In summary, the ancient 21-nt miRNAs are generated by DCL1 and recruited in AGO1 effectors, regulating targets by mRNA cleavage or translation inhibition, while non-conserved miRNAs could be processed by any member of the DCL family proteins into diverse-lengths, and hierarchically associated with different AGOs, consequently repressing targets via various modes (Figure 1; Table 1).

## miRNA FUNCTIONS IN PLANTS

Over the last 10 years, the number of annotated miRNAs has increased exponentially through deep sequencing of small RNA transcriptomes from diverse plant species and tissues, but only a few of them have been determined their functional roles. Here we summarize some of recent advances in knowledge on the associations of miRNAs and physiological processes, particularly focusing on the regulatory effects of the non-conserved miRNAs.

### BIOLOGICAL SIGNIFICANCE OF CONSERVED miRNAs

Features of conserved miRNAs lie not only in their conserved sequences but also in the analogous characteristics of their targets. During long-term evolution, the deeply conserved miRNAs have been integrated early in the regulatory networks to orchestrate plant developmental processes through control of a family of proteins. Despite this, some conserved miRNAs could also evolve lately to acquire novel targets and play alternative roles in specific lineages.

### ORIGINAL ROLES OF CONSERVED miRNAs

As stated above, diversification of conserved miRNA functions in different plants appears to be scarce perhaps owing to the long-term evolutionary selection of old miRNAs and targets (Chapman and Carrington, 2007; Voinnet, 2009; Axtell, 2013). The first evidence of the significance of miRNAs in plant came from two conserved miRNAs, miR319 and miR172 that affect leaf and flower morphogenesis by controlling TEOSINTE BRANCHED1/cycloidea/PCF (TCP) and Apetala2 (AP2) transcription factors (Palatnik et al., 2003; Chen, 2004). From then on, there has been an ever-growing increase in literature on ancient miRNAs targeting versatile transcription factors to influence essential signaling pathways in plant growth and responses to environmental stresses. Due to the space limitations of manuscript required, the conserved biological importance of original miRNAs are not discussed in detail here, and their discussion can be easily found in other frontier reviews (Voinnet, 2009; Rubio-Somoza and Weigel, 2011; Chen, 2012; Poethig, 2013).

### ALTERNATIVE ROLES OF CONSERVED miRNAs

In addition to controlling orthologous targets in various plants, several ancient miRNAs have been recently revealed to recognize non-conserved targets in specific plant species, thus playing alternative roles in plant growth.

Besides targeting growth-regulating factor (*GRF*) genes in flowering plants, miR396 has been found to affect leaf growth via the acquisition of a neoteric target (*bHLH74*) in Brassicaceae and Cleomaceae species (Debernardi et al., 2012). Not only *GRF* family gene mutants, but also loss-of-function of *bHLH74* plants displayed abnormal defects in leaf margin and vein pattern formation in *Arabidopsis* (Debernardi et al., 2012), suggesting spatiotemporal regulation of both ancient and newly acquired targets by miR396 is required for leaf development in Brassicaceae. Intriguingly, the efficiency toward *GRFs* could be improved by addition of a nucleotide to the miR396 at the expense of reducing activity toward *bHLH74* (Debernardi et al., 2012), indicating that the bulges present between miRNA and target pairs may result in differential target modulation effects.

WRKY transcription factors have been clearly known to play a role in plant responses to biotic and abiotic stresses. In sunflower (*Helianthus annuus*), *HaWRKY6* transcript contains a putative target site of miR396. Under high temperature or salicylic acid treatment, sunflower plants show opposite expressions of miR396 and *HaWRKY6*, suggesting a possible role of its recently evolved regulation by miR396 during early responses to high temperature (Giacomelli et al., 2012). Considering that transformation of sunflower is difficult, the authors used a heterologous system and found that expressing miR396-resistant *HaWRKY6* in *Arabidopsis* not only were phenotypically smaller and accumulated more anthocyanin, but also caused more lethal damage than control plants (Giacomelli et al., 2012), further demonstrating a role of miR396-regulated *HaWRKY6* in plants against heat stress. Like the case of miR396-regulated *bHLH74* and *WRKY*, which is not related to canonical target GRF transcription factors in *Arabidopsis* and sunflower, a *non-MYB* gene (*SGN-U567133*) encoding a small nuclear-localized protein with unknown function has been unexpectedly discovered as a novel target for miR159 in tomato (Buxdorf et al., 2010). *SGN-U567133* is primarily expressed in flowers may be due to that SlmiR159-mediated the cleavage of this target is remote in this tissues. Moreover, transgenic tomatoes overexpressing miR159-resistant version of *SGN-U567133* were observed with strong defects in leaf and flower morphological phenotypes (Buxdorf et al., 2010), suggesting that SlmiR159-mediated *SGN-U567133* transcript cleavage is critical for tomato development.

The biological consequences reflected above imply that the importance in acquisition of new targets by ancient miRNAs should not be overlooked. Even though a series of non-conserved targets of ancient miRNAs have been predicted by bioinformatics and some cleavage events indeed have been validated by genome-wide degradome sequencing efforts (Addo-Quaye et al., 2008; German et al., 2008; Wu et al., 2009b; Li et al., 2010), the biological significance of their interactions is far from known, urgently requiring to be further deciphered in the future.

### BIOLOGICAL SIGNIFICANCE OF NON-CONSERVED miRNAs

Unlike conserved miRNAs, because either abundance of young miRNAs is low or young miRNAs have restricted spatial or temporal expression patterns, they have ever been considered to be evolved from random sequences and conferred few functions. Nonetheless, some non-conserved miRNAs are evident from recent studies that they could also have functions, especially fine-tuning roles, in target regulatory networks of different plants. Discovery of specific mechanism and functions of non-conserved miRNAs over a large number of conditions has now become a completely fascinating topic of investigation in plant miRNAs.

### BIOLOGICAL ROLES OF NON-CONSERVED miRNAs IN DICOTS

The first experimental report about non-conserved miRNA functions came from miR824, a Brassicaceae-specific miRNA identified from *Arabidopsis*. miR824 is formed by the duplication of its unique target gene AGAMOUS-LIKE 16 (*AGL16*) and mediates *AGL16* transcripts digestion at the typical miRNA-cleavage region (Table 2; Kutter et al., 2007). Overexpressing miRNA-resistant *AGL16* but not wild-type *AGL16* significantly increases the density of higher-order stomata in *Arabidopsis*, suggesting that miR824-mediated *AGL16* expression is critical for the development of stomatal complex (Kutter et al., 2007). A recent study also revealed that alteration of *AGL16* transcription by interruption of miR824 activity may repress *FLOWERING LOCUS T* (*FT*) expression, thereby mediating control of flowering timing of in *Arabidopsis* (Hu et al., 2014). These reports suggest that Brassicaceae-specific miR824 is able to play two different fundamental roles during plant growth process. miR163 is a special 24-nt miRNA containing a bulge structure between the miRNA and miRNA* in *Arabidopsis*, and is thus produced by DCL1 rather than DCL3 (Table 2; Wu et al., 2010). miR163 is highly expressed in *A. thaliana* but is not detectable in *A. arenosa* and is repressed in resynthesized allotetraploids due to weaker transcriptional expression of miRNA gene as well as lower efficiency of post-transcriptional processing precursors in *A. arenosa* and resynthesized allotetraploids than in *A. thaliana* (Ng et al., 2011). Because of being transcribed from inverted repeats, miR163 precursors share high levels of sequence identity with their targets SABATH methyltransferase family protein genes (*FAMT* and *PXMT1*). In *mir163* mutants, the target expression and plant metabolites profiles are profoundly changed, indicating that miR163-mediated target regulation controls secondary metabolite biosynthesis (Ng et al., 2011). Notably, although miR163 and its targets can be induced by fungal elicitor alamethicin both in *A. arenosa* and *A. thaliana*, the induction level is much different in the two species. This phenomenon suggests that the variation in miR163-mediated metabolic pathway may be important for growth vigor and stress adaptation in *Arabidopsis* species and allopolyploids (Table 2; Ng et al., 2011). It is interesting to explore that whether miR163 activity is also involved in heterosis in other plants besides *Arabidopsis.*

**Table 2:** **Targets and functions of non-conserved miRNAs validated by ectopic-expression experiments in plants.**

miRNA	Length (nt)	Targets	Lineage	Action modes	Functions	Reference
miR163	24	*PXMT1, FAMT*	*Arabidopsis*	RNA cleavage	Metabolite biosynthesis	Ng et al. (2011)
miR173	22	*TAS1, TAS2*	*Arabidopsis*	RNA cleavage, phased-siRNA initiation	Uncharacterized	Montgomery et al. (2008); Felippes and Weigel (2009)
miR400	21	*PPR*	*Arabidopsis*	RNA cleavage	Heat tolerance	Yan et al. (2012)
miR444	21	*MADS57*	*Oryza sativa*	RNA cleavage	Tillering development, nutrition accumulation	Guo et al. (2013); Yan et al. (2014)
miR472	22	*CNLs*	*Arabidopsis*	RNA cleavage, phased-siRNA initiation	Pathogen resistance	Boccara et al. (2014)
miR482	22	*NBS-LRR*	Solanaceae	RNA cleavage, phased-siRNA initiation	Pathogen resistance	Shivaprasad et al. (2012)
miR820	21,22,24	*DRM2*	*Oryza sativa*	RNA cleavage, DNA methylation	Epigenetic silencing	Nosaka et al. (2012)
miR824	21	*AGL16*	*Arabidopsis*	RNA cleavage	Stomata development, plant flowering	Kutter et al. (2007); Hu et al. (2014)
miR828	22	*MYB2*	*Gossypium*	RNA cleavage, phased-siRNA initiation	Fiber development	Guan et al. (2014)
miR858	21	*MYB2*	*Gossypium*	RNA cleavage	Fiber development	Guan etal. (2014)
miR5200	21	*FTL1/2*	*Brachypodium*	RNA cleavage	Flowering initiation	Wu et al. (2013)
miR4376	22	*Ca^2+^-ATPase*	Solanaceae	RNA cleavage, phased-siRNA initiation	Flower and fruit development	Wang et al. (2011)
miR6019	22	*NB-LRR/LRR*	Solanaceae	RNA cleavage, phased-siRNA initiation	Pathogen resistance	Li et al. (2012)
miR6020	21	*NB-LRR/LRR*	Solanaceae	RNA cleavage	Pathogen resistance	Li et al. (2012)
miR7695	21	*NrampG*	*Oryza sativa*	RNA cleavage	Pathogen resistance	Campo et al. (2013)

Another interesting example is miR400, an intronic miRNA, which is co-transcribed with its host gene (*At1G32583*) in *A. thaliana*. As a result of heat-induced alternative splicing regulatory machinery, mature miR400 is repressed under heat stress with an increase in its primary transcript levels (Yan et al., 2012). Ectopic miR400 transgenic plants displayed more sensitive to heat stress than wild-type plants, suggesting an essential role of post-transcriptional regulation of miR400 in the acquisition of thermo-tolerance in *Arabidopsis* (Table 2; Yan et al., 2012). Similar splicing-regulated mechanism is also observed for miR842 and miR846, the two functionally related miRNAs involved in abscisic acid (ABA) response in *A. thaliana* and *A. lyrata* (Jia and Rock, 2013). These results implicate a more complex process for generation and regulation of young miRNAs than ancient ones.

The interaction of a 22-nt miRNA with a target mRNA may trigger secondary siRNAs in phase, which is able to amplify the effects of RNA silencing in plants. Moreover, this class of secondary siRNAs may also form non-cell-autonomous signals to silence other genes in *trans*. Consequently, it is reasonable that guiding target transcripts to generate secondary siRNAs appears to be an efficient approach for the enhancement of target mRNA degradation by miRNAs. miR173, a typical 22-nt miRNA arisen from fold-back precursors with asymmetric bulges, targets a non-coding RNA and has been characterized as an initiator of phased siRNA formation at *TAS1* and *TAS2* loci in *A. thaliana* and *A. lyrata* (Table 2; Chen et al., 2007, 2010; Montgomery et al., 2008; Felippes and Weigel, 2009; Cuperus et al., 2010; Wu et al., 2012; Yoshikawa et al., 2013). Although *pentatricopeptide repeat* (*PPR*) transcripts have been experimentally validated to be digested by miR173-triggered phased siRNAs for long time, the physiological effects of miR173 and miR173-triggered phased siRNAs are still unknown (Allen et al., 2005; Montgomery et al., 2008; Wu et al., 2012).

The first biological relevance of interaction between non-conserved miRNA and target through secondary siRNA signaling has been discovered in Solanaceae plants (Wang et al., 2011). Subject to post-transcriptional processing modulation, miR4376 randomly occurs in Solanaceae families and mediates auto-inhibited Ca^2^^+^-ATPase (*ACA10*) mRNA degradation and phased secondary siRNA formation from target transcripts (Table 2; Wang et al., 2011). In tomato, low abundance of ACA10-originated phased siRNAs is directly correlated with the limited accumulation of miR4376 in flowers and fruits, whereas high-expression of miR4376 in leaves leads to a decrease of *ACA10* with an increase in phased siRNA accumulation. Ectopic expression of *ACA10* with a mutated miR4376 target site in tomato specifically altered the flower morphology and fruit yield, supporting the significance of miR4376-mediated *ACA10* control in plant reproduction (Table 2; Wang et al., 2011).

When invaded by pathogens or insects, plants may change some endogenous gene expressions including miRNA accumulations to trigger resistance responses. Tomato miR482 and tobacco miR6019 are such representative species-specific miRNAs in immune response to pathogens (Li et al., 2012; Shivaprasad et al., 2012). As a member of 22-nt miRNA family, miR482 slices the mRNAs of nucleotide-binding site–leucine-rich repeat (NBS–LRR) disease resistance proteins with coiled-coil (CC) domains at their N-terminus, and subsequently triggers a class of secondary phased siRNAs dependent on RNA-dependent RNA polymerase 6 (RDR6) activities. These secondary siRNAs not only target their precursors but also act on other mRNAs of defense-related proteins, resulting in a regulatory cascade formed in plant defense systems (Table 2; Shivaprasad et al., 2012). If tomatoes are challenged with viruses or bacteria, miR482-mediated target mRNA decay and secondary siRNA production are able to be inhibited, and thereby a large number of NBS–LRR proteins are increased, directly giving rise to a defense response against pathogen attack (Shivaprasad et al., 2012). In tobacco, miR6019 cleaves Toll–interleukin-1 receptor–nucleotide binding–LRR (TIR–NB–LRR) immune receptor *N* gene transcripts and guides synthesis of 21-nt secondary siRNAs requiring on RDR6- and DCL4 activities in phase with the target site (Li et al., 2012). Transient expression of N-targeting miR6019 in *Nicotiana benthamiana* attenuates N-mediated resistance to tobacco mosaic virus (TMV), indicating that tobacco must block miR6019-mediated attenuation of *R* gene expression to avoid biotic incursion (Table 2; Li et al., 2012). Interestingly, another 21-nt miRNA, namely miR6020, also mediates *N*-transcripts degradation and resistance to TMV, but it is unable to induce secondary siRNAs attributed to the absence of asymmetric bulge in its precursor structures (Table 2; Li et al., 2012; Manavella et al., 2012; Fei et al., 2013). These results indicate the involvement of two parts of complicated non-conserved miRNA-mediated silencing systems for precise regulation of *R* gene expressions in counteraction of pathogen attack in Solanaceae plants.

In addition to perceiving specific pathogen effectors, the non-conserved miRNAs-mediated secondary siRNAs have also been explored their roles in signaling events triggered by pathogen-associated molecular patterns (PAMPs). In transgenic plants lacking miR472, an *Arabidopsis*-specific miRNA, recapitulates the basal immunity phenotype of *rdr6* mutant which exhibits enhanced basal resistance toward a virulent *Pseudomonas syringae* strain, suggesting that miR472 and RDR6-mediated CC-NB-LRRs (CNLs) silencing pathway may have checkpoints modulating PAMP-triggered immunity (PTI; Boccara et al., 2014). Furthermore, overexpression of miR472 could enhance the number of secondary siRNAs at CNLs and compromise the PTI response when plants are subject to a biotic environment, supporting an essential role of secondary siRNAs by miR472 in CNL modulation and disease resistance (Table 2; Boccara et al., 2014). Because excessive *R* gene multiplication may lose high fitness costs (Heil and Baldwin, 2002), these non-conserved miRNA-mediated repression of *R* gene amplification and diversification would be of great biological significance in plants benefiting substantial growth fitness and defense.

The phased siRNAs generated by non-conserved miRNA also participate in plant development. GLABROUS1 (GL1) is a member of R2R3 MYB family of transcription factor and promotes leaf trichome development in *Arabidopsis* (Pesch and Hulskamp, 2009). There are two *GL1* genes including *GhMYB2A* and *GhMYB2D* homeologs in cotton, an allotetraploid (*Gossypium hirsutum*, AADD). Intriguingly, both of two MYB2 transcripts could be targeted by miR828 and miR858 in *G. hirsutum*, but more secondary siRNAs could be produced from *GhMYB2D* loci than that from *GhMYB2A*, since the former gene expressed much higher than the latter during fiber initiation (Table 2; Guan et al., 2014).The authors found that only overexpressing natural *GhMYB2A* but not *GhMYB2D* complements the *gl1* phenotype, but if mutate the miR828-binding site or replace the downstream target sequence in *GhMYB2D*, it can also restore trichome development in *gl1* mutants because the ta-siRNA production are abolished from *GhMYB2D* transcripts. This observation thus illustrates a unique role of mirR828 and mir858 in functional divergence between target homeologous genes for cotton fiber development (Guan et al., 2014).

Above all, it is apparent that non-conserved miRNAs have diverse functions in multiple biological processes in dicotyledons. With more investigation of miRNAs from distinct lineages, more physiological implications of non-conserved miRNAs will be uncovered from dicots in the future.

### BIOLOGICAL ROLES OF NON-CONSERVED miRNAs IN MONOCOTS

Compared with non-conserved miRNA roles characterized from dicots, those from monocots are even more limited.

miR444, which targets a class of MADS transcription factors in rice plants, has been identified for nearly 10 years (Sunkar et al., 2005). Although miR444 is abundantly accumulated in diverse monocots, its biological roles are obscure until two exciting discoveries made recently (Wu et al., 2009b). On the one hand, miR444 modulates OsMADS57, the product of which interacts with a TCP-family transcription factor (TEOSINTE BRANCHED1, OsTB1), and acts on Dwarf14 (D14), the potential receptor of strigolactones to control the outgrowth of axillary buds and tillering in rice (Table 2; Guo et al., 2013). On the other hand, miR444 could simultaneously control four MADS-box genes, and may participate in ${\text{NO}}_{\text{3}}^{-}$ and Pi^–^ signaling pathways through alteration of lateral root architecture when rice are subject to detrimental nutrient environments (Yan et al., 2014). These findings suggest miR444 is able to play multiple roles in rice growth, and perhaps more additional roles of miR444 could be identified from further explorations in other monocots in addition to rice.

Excessive transposable elements (TEs) can destroy a eukaryotic genome; many organisms thus have developed diverse mechanisms to inhibit TE activities. Majority of transposons and repeat elements in plants have been clearly known to be silenced by the RdDM machinery; however, how the components in the RdDM pathway are regulated remains poorly understood. miR820 is a species of 21-, 22-, and 24-nt small RNAs derived from a class of CACTA DNA transposon transcripts, potentially targeting a *de novo* DNA methyltransferase gene *DRM2* at both transcriptional and post-transcriptional levels (Table 2; Wu et al., 2010; Nosaka et al., 2012). The ectopic expression level of the *OsDRM2:GFP* fusion gene with an intact miR820 recognition site is observed much lower than that of genes with synonymous mutations, indicating that *DRM2* is indeed negatively regulated by miR820 in rice (Nosaka et al., 2012). Nevertheless, there is no clear inverse expression relationship between the levels of miR820 and targets, suggesting that miR820 may only reduce the amount of *DRM2* expression but may not abolish it completely (Nosaka et al., 2012). In transgenic plants showing *DRM2* RNAi and pre-miR820 overexpression, several transposons are *de novo* activated due to the decrease of DNA methylation status in *cis* (Nosaka et al., 2012). Together with the observation that the nucleotide sequence of miR820 and its recognition site within the target gene is co-evolved, these findings demonstrate that miR820-mediated *DRM2* modulation is essential for the maintenance of low transposon activities in rice (Nosaka et al., 2012). Future work will be needed to dissect miR820 expression pattern to define when and where rice accumulates more miR820 to enable transposon released.

Monocots have 24-nt secondary siRNAs in addition to 21-nt siRNAs. In rice, miR2118 and miR2275 are involved in initiation of these two classes of siRNAs, respectively (Song et al., 2012). Intriguingly, both miR2118 and miR2275 as well as the 21- and 24-nt secondary siRNAs have been found preferentially expressed in panicle and stamens, suggesting that these sRNAs may functionally contribute to reproductive organ development in rice (Song et al., 2012). Nevertheless, experimental genetic studies by overproducing and silencing miR2118 and miR2275 activities are required to further clarify their respective roles in rice panicle and stamens formation.

Through high-throughput sequencing, Campo et al. (2013) identified a novel DCL4-dependent miRNA designated miR7695 from blast fungus-treated rice plants. This miRNA targets an alternatively spliced transcript of *Nramp6* (*natural resistance-associated macrophage protein 6*; Table 2; Campo et al., 2013). Compared with non-transformed plants, the percentage of *M. oryzae* diseased leaf area exhibited much lower than that in miR7695 overexpressing transgenic plants, suggesting that miR7695 contributes to plant resistance to fungal pathogen (Table 2; Campo et al., 2013). Interestingly, miR7695 can only mediate cleavage of one *Nramp6* splicing variant but not of all transcripts, representing a complicated regulatory machinery of plant immunity requiring in miRNA-mediated gene regulation (Campo et al., 2013).

In addition to rice, the physiological connections between non-conserved miRNAs and plant growth were also investigated in other monocots. With the attributes of small stature, short generation time, and small genome, *Brachypodium* is an ideal biological research model for temperate cereals such as wheat and barley, and has been implicated in occupying special genetic pathways for flowering time control compared with eudicotyledons and rice (Higgins et al., 2010). Although numerous lineage-specific miRNAs has been identified from *Brachypodium* (Wei et al., 2009; Zhang et al., 2009; Jeong et al., 2013), none of their roles are clear until a recent study by us. We characterized a Pooideae-specific miRNA (miR5200) targeting two *FLOWERING LOCUS T* orthologous genes in *Brachypodium* in a mRNA cleavage manner. miR5200 has similar tissue expression patterns with *BdFTL1* and *BdFTL2* mRNAs that distinctively accumulate in plant leaves but are absent in shoot apices, thus spatially conferring regulatory activity to the targets (Table 2; Wu et al., 2013). Ectopic expression of miR5200 in transgenic *Brachypodium* plants significantly decreases *BdFTL1* and *BdFTL2* as well as downstream gene expressions and thereby severely delays flowering time. Furthermore, miR5200 is sensitive to photoperiod, exhibiting highly accumulated when *Brachypodium* plants are grown in short-day (SD) conditions but dramatically repressed in plants grown under long-day (LD) environments. Interestingly, we found that the histone status of H3K27me3 and H3K4me3 in miR5200 precursor genes could change according to day-length, resulting in distinct expression of miR5200 for photoperiodic control of flowering under SD and LD conditions (Wu et al., 2013). Blocking of miR5200 activity in plants through a targeted mimic approach specifically alters flowering time in SD conditions but not in LD conditions, indicating that miR5200-mediated regulation of *FT-like* gene plays an important role in flowering initiation in *Brachypodium* (Table 2; Wu et al., 2013). Because photoperiodic regulation of miR5200 appears to be prevalent in Pooideae plants (Wu et al., 2013), it may be feasible to artificially manipulate miR5200 behavior to improve agricultural traits of wheat and barley for wider adaptations in the future.

## CONCLUSION AND REMARKS

The extraordinarily fast development of high-throughput sequencing technologies has enabled scientists to uncover a large number of novel sRNAs in recent days. Countless bioinformatics data of miRNAs have been released and become freely available in updated miRNA databases, providing significant basis for miRNA functional investigations. However, compared to conserved miRNAs whose functions were extensively explored and validated, non-conserved and lineage-specific miRNAs have been ignored in biological studies even if they account for the major part of miRNA families.

Encouragingly, growing bodies of evidence continuously illustrate that non-conserved miRNAs are also regulators of gene expression and sometimes function as crucial determinants of plant morphology as well as defense against pathogen disease (Zhai et al., 2011). Unlike ancient miRNAs, which usually serve as transcription factor modulators, non-conserved miRNAs have broader predicted targets, including active enzymes and physiological proteins involved in adaption to multifarious conditions.

Besides of guiding gene transcript digestion and protein translation inhibition, non-conserved miRNAs can control targets through inducing secondary siRNA formation or trigging DNA methylation. miR173 in *Arabidopsis*, miR2275 in rice, and miR482 in tomatoes are typical examples of non-conserved miRNAs that mediate phased siRNA initiation for the enhancement of target regulation (Shivaprasad et al., 2012; Song et al., 2012; Fei et al., 2013). Although it is clear that lmiRNAs act on target DNA in rice, their biological role and phenotypic relevance in epigenetic modeling have not yet been illustrated (Wu et al., 2010).

Moreover, regulatory modules composed by multiple conserved miRNAs and targets have been identified to be involved in diverse interconnected biological programs, including flowering maturation, flowering initiation, lateral organ growth, nodule development, senescence, and phytohormone signaling (Wu et al., 2009a; Koyama et al., 2010; Gutierrez et al., 2012; Mao et al., 2013; Rubio-Somoza and Weigel, 2013; Curaba et al., 2014; Spanudakis and Jackson, 2014), implying that there also may be conceivably coordinated relationships between conserved and non-conserved miRNAs in diverse genetic pathways (Jin et al., 2013). For instance, whether miR156 and miR172 affect miR5200-controlled FT module in temperate cereals, whether miR159-regulated MYB transcription factors can influence miR842 and miR846 expressions in *Arabidopsis* plants subject to ABA, whether miR156-SPLs module is able to upstreamly affect primary miR444 transcripts to control tillering in rice, and whether miR393 affects the accumulation of defense miRNAs required for secondary siRNA generation in biotic-stress responses through auxin signaling pathway are interesting to be investigated. Thus, it is important to explore and characterize more possible mechanistic significances of non-conserved miRNAs at first in different plant species.

Genetic engineering is thought to be the most rapid way to resolve the challenges in modern agriculture nowadays. Because plenty of miRNAs have been found to be involved in plant morphogenesis, researchers are beginning to take advantage of operating miRNA actions to improve plant architecture for increasing crop yield. miR156-modulated SPL14 has been implicated in the semi-dominant quantitative trait locus termed as *IPA1* in rice (Jiao et al., 2010; Miura et al., 2010). Introduction of a point mutation in *SPL14* could interrupt miR156-mediated regulation of *SPL14* and generated an optimized rice plant with few unproductive branches, enhanced lodging resistance, and improved grain yields (Jiao et al., 2010). Laccase-like proteins (LACs) are regulated by miR397 and are involved in brassinosteroids (BR) signaling. Overexpression of miR397 in rice dramatically represses *LAC* expressions, simultaneously enlarges grain size, and promotes panicle branching, thus significantly increasing grain production (Zhang et al., 2013). Now that increasing rice yield through miRNA actions is practical, it will be promising to improve agricultural traits of crops more economically by fine-tuning the regulatory module between non-conserved miRNA and targets in the future.

### Conflict of Interest Statement

The authors declare that the research was conducted in the absence of any commercial or financial relationships that could be construed as a potential conflict of interest.
